# An Analysis of Hospital Accreditation Policy in Iran

**Published:** 2017-10

**Authors:** Taraneh YOUSEFINEZHADI, Ali Mohammad MOSADEGHRAD, Mohammad ARAB, Mozhdeh RAMEZANI, Ali AKBARI SARI

**Affiliations:** 1. Dept. of Health Management and Economics, School of Public Health, Tehran University of Medical Sciences, Tehran, Iran; 2. Dept. of Community Medicine, School of Medicine, Iran University of Medical Sciences, Tehran, Iran

**Keywords:** Hospital, Accreditation, Policy analysis, Iran

## Abstract

**Background:**

Public policymaking is complex and lacks research evidences, particularly in the Eastern Mediterranean Region (EMR). This policy analysis aims to generate insights about the process of hospital accreditation policy making in Iran, to identify factors influencing policymaking and to evaluate utilization of evidence in policy making process.

**Methods:**

The study examined the policymaking process using Walt and Gilson framework. A qualitative research design was employed. Thirty key informant interviews with policymakers and stakeholders were conducted. In addition hundred and five related documents were reviewed. Data was analyzed using framework analysis.

**Results:**

The accreditation program was a decision made at Ministry of Health and Medical Education in Iran. Many healthcare stakeholders were involved and evidence from leading countries was used to guide policy development. Poor hospital managers’ commitment, lack of physicians’ involvement and inadequate resources were the main barriers in policy implementation. Furthermore, there were too many accreditations standards and criteria, surveyors were not well-trained, had little motivation for their work and there was low consistency among them.

**Conclusion:**

This study highlighted the complex nature of policymaking cycle and highlighted various factors influencing policy development, implementation and evaluation. An effective accreditation program requires a robust well-governed accreditation body, various stakeholders’ involvement, sufficient resources and sustainable funds, enough human resources, hospital managers’ commitment, and technical assistance to hospitals.

## Introduction

The health system of Iran has been improved during the last three decades. At national level, the Ministry of Health and Medical Education (MOHME) is responsible for policy-making, funding, planning, leading, coordinating, and evaluating the healthcare services provided by a variety of public and private healthcare organizations and institutes. This responsibility has been delegated to the Universities of Medical Sciences and Health Services (UMSs) at provincial level. UMSs are also responsible for educating and training human resources needed for delivery of healthcare services (1).

Public and private hospitals provide the secondary and tertiary healthcare services. All hospitals must have a license for operation. Certain structural and procedural standards have to be met to receive such a license. The license has to be renewed every five years. In addition, hospitals have to go through an annual accreditation process to be eligible for providing healthcare services. Besides, un-noticed visits can be done by the health authorities for the aim of continuous quality and safety assessment. The assessment and evaluation are performed by accreditation office at UMSs. At national level, the Office for the Accreditation of Healthcare Institutions is responsible for policy making, planning and leading the national hospital accreditation program. Hospitals are reimbursed on a fee for service base. The hospital tariff is based on its accreditation grade. The higher the hospital grade, the higher the tariffs it charges patients (2).

The first hospital evaluation system in Iran was established in 1962. Limited number of structural standards was used for hospital evaluation. In 1997, more structural and procedural standards were added to the hospital evaluation checklists and several specialized surveyors have been recruited to assess diverse areas of the hospitals’ activities. Finally, in 2012, the hospital evaluation system was upgraded to the hospital accreditation system (3).

Hospital accreditation is “a systematic external evaluation of a hospital’s structures, processes and results (outputs/ outcome) by an independent professional accreditation body using pre-established optimum standards” (4). The objectives of a hospital accreditation system are to assess the quality and safety of patient care and to encourage continuous quality improvement through using optimal yet achievable standards. It helps improve public confidence in healthcare services provided by hospitals (5). An independent national body normally assesses the health-care organizations’ compliance with predetermined input, process and output/outcome standards and gives the successful candidate organization an accreditation certification for a period of two to three years (3, 4).

Historically, the American College of Surgeons firstly developed accreditation standards for surgical training in 1917 which later led to the establishment of the Joint Commission on Accreditation of Healthcare Organizations (JCAHO) in 1951 (6). During the 1980s and 1990s the number of accreditation bodies increased considerably in the world (7).

Accreditation helps improve hospitals’ systems, processes, operational effectiveness and outcomes (7,8). It provides a vision of sustainable quality improvement (9, 10), supports the effective and efficient use of resources (11), promotes capacity-building, professional development and organizational learning (12), strengthens interdisciplinary team effectiveness (13, 14), and improves communication and collaboration internally and externally (15, 16). The accreditation program promotes a quality and safety culture (17, 18), encourages the sharing of policies, procedures, and best practices among health care organizations (19), mitigates the risk of adverse events (20, 21), leads to the improvement of internal practices (13), increases compliance with safety and quality standards (22, 23), and sustains improvements in quality and organizational performance (15,21, 24). Hospital accreditation improves patients’ health outcomes (25, 26), perception of quality care (15, 22), and satisfaction (8) and contributes to improved staff working conditions and quality of life (27) and increased job satisfaction (22, 28). An accreditation certification demonstrates credibility and a commitment to quality and accountability (8, 15, 23, and 29) and improves the organization’s reputation among end-users (15).

Health policy includes plans, and actions for achieving health care goals through defining a vision for the future, establishing goals, objectives and targets, setting priorities and specifying different groups’ roles (30). Policy making is a process of agenda setting (problem identification), policy formulation, policy implementation and policy evaluation (31). An evidence-informed health policy has a crucial role in improving health, reducing health inequities and contributing to economic development (32). Studying the role of evidence in policymaking allows a better understanding of the contribution of research in the formulation of policies and identifying influencing factors (33). However, research evidence is still underutilized in policy-making in the Eastern Mediterranean (34). Health policy analysis helps understanding the grounds for a past policy’s success or failure that could be useful for planning future policies (35). Evidence from health policy analysis can potentially increase policy impact and provide information that may assist with the allocation of scarce resources. Health policy analysis assists policy makers to improve the chances of successful implementation of future policy by revealing opportunities where enhancements to policy documents may be made. Such enhancements may be added to future policy documents or potentially to the original documents if applied before the policy is finalized (36).

This study focusing on policy analysis of hospital accreditation in Iran aims to generate in-depth insights on the process of policy making, to identify factors influencing policymaking, to assess the extent that evidence is used, to examine the impact of hospital accreditation policies and to reflect on lessons learned for informing future public policymaking. Such a study provides insights for structuring the decision-making process.

## Methods

**Fig. 1:**
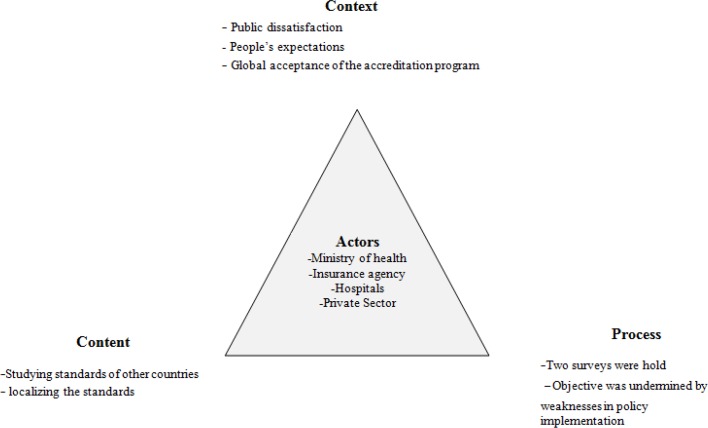
Iran’s hospital accreditation policy analysis using Walt and Gilson framework

This qualitative study explored the “Iranian Hospital Accreditation (IHA)” policy making process using Walt and Gilson triangle framework (Fig. 1).

Walt and Gilson (1994) developed a policy analysis triangle framework incorporating context, actors, process and content concepts specifically for healthcare sector. The triangle framework allows the analysis of the contextual factors such as social, economic, and political factors that influence the policy, the process by which the policy was initiated, formulated, developed, implemented and evaluated, the objectives of the policy and the actors involved in the policy making (37).

Furthermore, Kingdon’s (1984) Multiple Streams Framework (MSF) was used to analyze further the policymaking process of the national hospital accreditation in Iran. Kingdon using the multiple-streams theory in agenda setting argues that the public policy process has a random character, with problems, policies and politics flowing along in independent streams. A “policy window” opens only when these three independent streams converge, propelling governments to act. The problems stream contains the broad problems facing societies. The policy stream refers to the set of policy alternatives that researchers and other stakeholders propose to address problems. The politics stream consists of political transitions, national mood, elections, or pressure from interest groups (38).

Thirty face to face semi-structured interviews were conducted between April and September 2015. A purposeful sampling method was used (Table 1). Interviews were digitally recorded after obtaining informed consent and each lasted between 45 and 60 minutes. Two interviewees refused to be tape-recorded. Collected data were coded and organized based on key themes (identified through framework analysis) using MAXQDA software (version 10).

**Table 1:** Interviewees Characteristics

***Position/Organization***	***Number***
Faculty member	3
Medical council	1
Expert of Ministry of Health	6
Insurance agency	2
Hospital manager	3
Policy maker	4
Private sector	1
Surveyor	10
Total	**30**

In addition, related documents were reviewed and analyzed to explore the role of evidence in the policy making process and to validate the information obtained from interviews. Hundred and five documents were obtained dating from 1985 to 2015 including official letters, legislations, law decrees, directives, reports and minutes of meetings of Ministry of health. The review of documents was conducted by a reviewer and checked by a second reviewer. The document review was guided by a protocol adapted from Hanney et al. (32).

## Results

### Context

Since 1997, all Iranian hospitals were evaluated annually using a checklist covering more structural standards in fifteen domains including management, medical staff, nursing staff, administrative and support staff, safety equipment, medical equipment, non-medical equipment, medical records, education and research, religious and humane values, patients satisfaction, hospital committees, sanitation, physical structure and some limited quality indicators. Less attention was paid to procedural and outcome standards in such a mandatory program.

On-site surveys were conducted by a team of 3–10 surveyors each focusing on specific domains of the standards. The Accident and Emergency (A&E) department of the hospital was evaluated first. If the A&E department achieved lower score than the rest of the hospital’s departments and wards, the grade of the A&E department was declared as the hospital grade. A range of five evaluation grades including excellent, one, two, three, and non-standard were applied. The tariff of hospitals was linked to their evaluation grade. Thus, there was an incentive for hospital managers to improve hospital performance, so, they could charge patients a higher tariff. For those hospitals that achieved grade non-standard, they have been given a time of three months to improve their performance and solve the problems identified during the evaluation process.

The development of the hospitals’ accreditation policy was mainly due to the failure of the hospital evaluation system in providing a clear comprehensive and valid picture of hospitals’ performance specially regarding to the quality and safety of services. Hospitals that achieved higher grades had still problems with the quality and safety and patients were not satisfied with them. The overall awareness of the people about quality and safety had increased as so as their expectation. A senior official at health insurance organization said: “*more studies on patients’ rights are conducted. The health system should be more accountable for the safety and quality of healthcare services provided at hospitals. The aim of introducing accreditation was to enhance the hospital’s responsiveness. The hospital evaluation system was not very effective at that time”*.

There was a pressing need to upgrade hospital standards and include more comprehensive structural, procedural and outcome standards. Some hospitals voluntarily used the industrial ISO and EFQM models for self-assessment and even applied for their certifications. The fast growing recognition of hospital accreditation worldwide particularly in the EMRO region’s countries such as Egypt, Lebanon and Jordan encouraged some Iranian hospitals to apply for international accreditation programs such as Accreditation Canada International. As the accreditation model was specifically developed for the health sector, it was decided to use an accreditation model for the country. Thus, the authorities at ministry of health considered developing a national accreditation model for Iranian hospitals and a few researches have been conducted on hospital accreditation models of US, France, Lebanon and Egypt.

The existence of a structure for hospital evaluation system at MOHME and UMSs and familiarity of hospital managers and employees with the evaluation system facilitated the execution of the accreditation program.

### Content

The objectives of initiating an accreditation program were to use more comprehensive structural, procedural and outcome standards to enhance the quality and safety of hospital services, to respond better to the clients’ needs, to improve hospitals’ key performance indicators, to reduce costs, to improve clients’ satisfaction and to develop health tourism.

Reviewing the accreditation standards of some pioneer countries such as USA and France, as well as some Middle Eastern countries such as Egypt and Lebanon and holding some focus group discussion meetings with evaluation and accreditation experts helped adopt, develop and adapt accreditation standards for Iranian hospitals. Departmental approach was used to develop hospital accreditation standards. Overall 8104 criteria were used for the accreditation of 37 hospital departments and wards. After the first round of national hospital accreditation survey in 2013, some changes have been applied to the content of the standards based on the comments received from the hospital managers and employees and surveyors. As a result, some criteria were merged together and 2157 criteria for accreditation of 36 departments of the hospital were included in the accreditation program. These standards mainly focused on structures and processes rather than outcomes.

### Actors

The accreditation program was a decision made at the MOHME. Some people who were acquainted with the hospital accreditation program and had good relationship with the authorities at the hospital evaluation department of MOHME had critical role in initiating the program there. Then, some hospital accreditation consultants were invited to help develop the standards and running the program. The concept of hospital accreditation, its differences with the previous evaluation system and its goals were explained to the other departments of the ministry of health to get their support. Insurance companies well accepted the program as they assume the accreditation program could improve the quality and safety of hospital services better than the old evaluation system. They believed that the results of the accreditation program could be more reliable as it uses comprehensive set of standards.

Nevertheless, there was some resistance mainly from the private hospitals. They got used to the previous evaluation system and they found the number of the standards and criteria as the main barrier to implement them. They found it difficult to coordinate hospital resources to implement 8104 accreditation criteria. The Office for the Accreditation of Healthcare Institutions (OAHI) finalized the first draft of the standards and piloted it in eight hospitals and as a result the standards were modified using the comments received. Then, the standards were announced to all hospitals including private hospitals. Consequently the resistance was gradually decreased.

### Policy making process

#### Agenda Setting

Too much emphasize on the structural standards, lack of procedural and outcome standards, the reports of medical errors and lack of public satisfaction of the quality of hospital services were among the main problems of the hospital evaluation system (problem stream). The political stream had been created as the authorities at the MOHME demanded a change in the hospital evaluation system to reduce public dissatisfaction. The quality and safety of hospital services was a priority for top managers at MOHME. Hospitals were asked to apply measures to improve the quality and safety of their services. Therefore, the hospital evaluation system should be changed to embed quality and safety standards.

One alternative to solve the problem was considering other evaluation models such as ISO and EFQM. However, the success of the accreditation model worldwide and its healthcare characteristics urged the authorities to consider it as an applicable solution for solving the problem. Accordingly, a comparative study of several countries’ accreditation program began to help choosing a hospital accreditation model for Iran.

Some officials suggested that an accreditation council comprising of representatives from government regulatory agencies, professional organizations, practitioners, and the public should be created to govern the accreditation program. The idea was not accepted. It was assumed that as the program is new, including many parties in the governing body of the accreditation program make it complicated and as a result the resistance would be increased. It was decided to make the program mandatory and to be carried out annually by the MOHME with the cooperation of UMSs. Subsequently, the accreditation policy window has been opened and set the hospital accreditation program in the agenda.

### Policy formulation

The hospital accreditation standards were formulated in six phases. In phase one, a study of the hospital accreditation programs and standards of US (JCI), France, Egypt and Lebanon was conducted in 2007. In the second phase, the necessary local standards were added to the list of standards. In the third phase of the project, a departmental model was used for the hospital standards and the first draft of the standards was prepared. In the fourth phase, a poll was conducted and different symposiums were held on the first draft of the standards to know the opinions of different groups of experts. Standards were piloted in eight hospitals in the fifth phase to pinpoint the probable problems. In other word, the performance of these hospitals was evaluated using the new set of hospital accreditation standards. Finally, in the sixth phase, after holding a number of meetings with the representatives from various departments of MOHME, UMSs and academic associations, the final copy of the accreditation standards were formulated and communicated to hospitals for implementation. The entire process of standards development, obtaining feedback, pilot testing, refinement, and final printing took three years.

### Implementation

The cascading training for implementing hospital standards was organized to be conveyed from the OAHI to UMSs and then to the hospitals. The first accreditation survey was done in 2012. There were no specific criteria for appointing surveyors. More than 700 surveyors were chosen from the curative affairs department of UMSs and trained. An on-site survey team consisted of about 20–25 surveyors who were chosen from UMSs staff in some specific fields (e.g. medicine, nursing, pharmacy, radiology, health management, etc.). At least one physician and a nurse were included in the team. The full accreditation surveys involved an intensive process of reviewing documents and conducting site tours, observations, and staff and patient interviews. Trained surveyors performed the surveys. The surveyors submitted the hospitals’ scores on the devices prepared for them through the portal of ministry of health. The formal report of the hospitals’ scores and grades were sent to the UMSs to communicate to the hospitals. Hospital grades were Excellent, One plus, One, Two, Three and non-accredited. Almost 71.4 percent of hospitals achieved grade one and over.

The implementation of the accreditation program and standards was with some difficulties. Insufficient personnel to implement the standards were the main challenge of the hospitals. Poor hospital management and leadership commitment, lack of time, and lack of physicians’ involvement were also inhibiting proper implementation of the accreditation standards. Some problems with the contents of the standards and criteria were also mentioned by the interviewees such as too many standards, insufficient outcome standards, ambiguity of standards and criteria, imbalance of the standards of various departments of the hospital, and the scale of scoring. Lack of coordination between the accreditation program and other programs in the Ministry of Health also caused problems for hospitals. As a result, hospitals had to apply many standards posed by various departments of the ministry of health.

Furthermore, surveyors were not well-trained and had little motivation for their work. The surveyors had to do their routine duties in UMSs in addition to the hospital surveys. They were overloaded and less motivated. As a result, there was a lack of reliability in the accreditation process. Surveyors emphasized more on the documentations during the first round of the accreditation survey. This put more financial burden on the hospitals to prepare the documents and took too much time of staff specially the nursing personnel. Lack of a national hospital policies and procedures forced hospitals to prepare all those policies and procedures themselves which took too much of the time of hospital personnel. One hospital manager commented: “*The personnel are still unaware of the goals of the* [accreditation] *program. There are not enough personnel in nursing and para-clinical wards and there is no possibility to recruit more staff. Personnel are under pressure which in turn lowers the quality of services”.*

The second round of the accreditation program was carried out in 2014 during which the number of standards and criteria were reduced and the surveyors received new trainings. Reducing the number of standards and criteria and putting more emphasis on the process of implementing the standards rather than documentation solved some of the previous mentioned problems. However, some problems such as standards ambiguity, imbalanced distribution of the standards and the scale of scoring were remained unsolved. Hospitals’ managers and staff complained also about the differences in the surveyors’ opinions. Therefore, two clinical and administrative senior surveyors from other provinces accompanied the surveyors team and took the lead in the second round of hospital accreditation. After the second round of the accreditation survey a five category scale of excellent, one, two, three and non-accredited were used for grading hospitals.

### Evaluation

Some measures were taken at the MOHME to evaluate the process of the accreditation program. For instance, the education and training programs of UMSs for hospitals on accreditation, and the process of hospital accreditation upon the request of the hospitals were evaluated by OHOA. Hospitals that achieved the excellent grade were reassessed. Hospitals’ managers and staff and surveyors could raise their concerns on accreditation through OAHI website. UMSs were also asked to send their concerns and suggestions to OAHI. After the second accreditation survey in 2015, the opinions of all hospital managers on accreditation standards, methods, implementation and effects were solicited through a national survey. Five hundred and forty seven hospitals participated in the survey. However, there was no attempt to evaluate the outcome of the hospital accreditation due to the difficulties in measuring and collecting hospital key performance indicators. One senior official from the Ministry of Health indicated: “*Unfortunately, there is a lot to do for the outcome indicators. Hospitals should learn to measure the indicators. Until then, they will only work on the procedural indicators”.*

## Discussion

The hospital accreditation policy in Iran was well-intentioned aiming to enhance the quality and safety of services provided in Iranian hospitals due to the shortcomings of previous hospital evaluation system. Lack of procedural and outcome standards were the main problems of the hospital evaluation system. There was a pressing need to upgrade the hospital evaluation system and optimize the standards and include more quality and safety standards. Many healthcare stakeholders were involved in the hospital accreditation policy making process and evidence from the leading countries was used to guide policy development. However, benchmarking just a few countries’ hospital accreditation programs could not provide enough evidence for developing a comprehensive set of standards. As a result, the hospital standards and criteria in Iran were not inclusive and less attention was paid to the outcome standards. Furthermore, frequently quick revision of accreditation standards caused many problems for hospitals.

The hospital accreditation program did not achieve the intended benefits due to the shortcomings in policy implementation. Insufficient resources and information to implement accreditation standards in hospitals were the main barriers in policy implementation. Furthermore, surveyors were not well-trained, had little motivation for their work and didn’t use the same approach in hospital evaluation and accreditation.

Overall, the responsibility of hospital accreditation in Iran is laid in the hands of OAHI at the ministry of health. In other word, a governmental institute governs hospital accreditation program in the country. About 80 percent of Iranian hospitals are owned by the government. Thus, if the accreditation body’s structure and governance is not independent, there is a chance of having biased results. Smits and colleagues (2014) believe that low and middle income countries lack the financial capacity and resources to set up independent free-standing accreditation bodies. As a result, the accreditation bodies in these countries are governmental (39). To be more independent, OAHI should establish an accreditation council and three independent committees to deal with standard development, accreditation, and appeals (4).

**Fig. 2:**
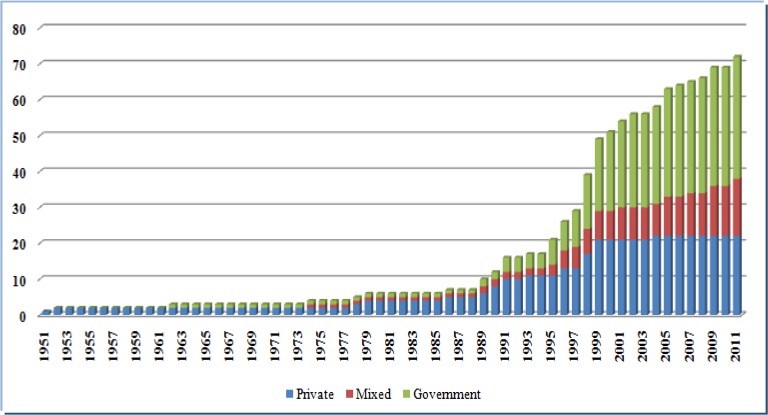
Hospital Accreditation Organizations, 1951–2011(47)

Worldwide different approaches are used for administration of the hospital accreditation program. For instance, the accreditation body in countries such as Lebanon, Italy, Scotland, England and France, is a governmental institute. In contrast, in the United States and Canada, private independent agencies manage the accreditation program (6). In Malaysia, the accrediting body was formed in collaboration with the Ministry of Health, the Private Hospital Association, and the Medical Association (39). Nevertheless, the important thing is engaging all stakeholders in the accreditation program. A participative approach should be used in the developing the hospital accreditation program and standards. Key stake-holders such as UMSs, medical insurance companies, medical and nursing associations should play a critical role in the implementation of the accreditation program (40). Accreditation is a strategy for improving the quality, safety and effectiveness of healthcare services. Therefore, the governments prefer to take responsibility and make it compulsory for all hospitals (Fig. 2).

Iran hospital accreditation program faced some challenges regarding to the content of the standards and the way those standards have been implemented (3, 4). Too many standards, insufficient outcome standards, ambiguity of standards and criteria, imbalance of the standards of various departments of the hospital, and the scale of scoring, insufficient personnel to implement the standards, poor hospital management and leadership commitment, lack of time, and lack of physicians’ involvement were inhibiting proper implementation of the accreditation standards.

World Health Organization (WHO) has mentioned some challenges for hospital accreditation in Eastern Mediterranean such as giving a precise value or numerical score to findings, having standards for a few hospital units rather than all hospital services, lack of an inter-institutional and independent national commission on hospital accreditation, multiple accreditation entities, competing among each other, and setting different standards, priorities and fees, and confusing licensing with accreditation, misperception of the role of surveyors (41).

The Iranian hospital accreditation is in its infancy. We cannot expect the same results of well-established accreditation programs like what we see in US, Canada and Australia which have an experience of 65, 57 and 42 years (5). Their standards revised several times. For example, JCAHO reviews its standards every 2 years to get advice from experts, service providers and customers. In Canada standards are also developed regularly using the literature review, consultation with experts, health care professionals, customers, academics and policy makers, group discussion and piloting of hospital standards (14). Thus, the Iranian hospital standards should be gradually developed and customized with the hospitals’ resources and capacities. The Iranian hospitals cannot easily meet the standards of Joint Commission on Accreditation America, the 2014 version. The triad of structure, process and output/outcome was considered in the first version of hospital standards. However, more weight has been given to the structural and procedural standards (42). More outcome based standards should be added to assure the quality, safety and effectiveness of hospital services (43, 44). Analysis of the data from two rounds of accreditation surveys in Iran and various stakeholders concerns and expectations should be incorporated in the process of reviewing the standards.

Some policy options are proposed for improving the implementation of hospital accreditation program. International Society for Quality in Healthcare (ISQua) has some basic requirement for the healthcare accreditation programs including the accreditation body, standards and surveyors training, which can be used for benchmarking and improving the Iranian hospital accreditation program (17). Therefore, the OAHI, the accreditation standards and its surveyor training programs should be accredited by ISQua to increase the value, credibility and reliability of its evaluations.

The structural, contextual and procedural changes involved in the accreditation put too much stress on hospital managers and employees. Stress creates physical and mental problems for employees and negatively affect their quality of life (45) and performance (46, 47). Hospital managers have a crucial role in the success of change programs (48). Hospital top managers should be justified about the benefits of the accreditation to guarantee their commitment and leadership for the quality transformation. They should provide necessary resources for improving the quality, safety and effectiveness of hospital services (49, 50). Physicians should get trained and be involved more in the process of implementing standards.

Hospital surveyors should be selected and recruited based on specific and obvious job description and person specification and should pass a comprehensive training course to be eligible for the surveys. Training surveyors helps reduce the inconsistencies among them during the accreditation process (4). Capable and professional surveyors are crucial in an accreditation program to conduct the survey in a standard manner. In Iran, surveyors were initially selected through nomination by the treatment department of UMSs and were paid less relative to the level of work demanded. The same problem was reported in Zambia which resulted in surveyors’ low commitment and high turnover (51). Surveyors should give constructive suggestions to hospitals on how to achieve the accreditation (4). A descriptive report of the hospitals performance based on the results of the surveys should be provided for the hospitals. More technical assistance should be offered to the hospitals by UMSs. It is not necessary to conduct the accreditation survey every year owing to its cost. It is suggested to consider a time frame of two years for surveys.

## Conclusion

This study highlighted the complex nature of policymaking cycle and various factors influencing policy development, implementation, and evaluation. The underlying philosophy of the hospital accreditation as a strategy for quality and safety improvement was not fully congruent with the Iranian context. Hospital managers treated the accreditation as the final goal instead of a means to achieve service quality. An effective accreditation program requires a robust well-governed accreditation body, sufficient and sustainable funds, enough human resources, management commitment, education and training, and technical assistance to hospitals. Various stake-holders’ involvement should be encouraged in the governance of the accreditation program. Appropriate surveyors should be selected and trained professionally to ensure inter-rater reliability among them. On time, feedback about the accreditation survey and ongoing technical assistance should be provided for hospitals on standards implementation.

## Ethical considerations

Ethical issues (Including plagiarism, informed consent, misconduct, data fabrication and/or falsification, double publication and/or submission, redundancy, etc.) have been completely observed by the authors.
